# Supplementary material for: Trends of Lipophilic, Antioxidant and Hematological Parameters Associated with Conventional and Electronic Smoking Habits in Middle-Age Romanians
Source: J Clin Med. 2019 May 12;8(5):665. doi: 10.3390/jcm8050665 (PMC6571835; doi:10.3390/jcm8050665)
Supplement: Supplementary file 1 [file jcm-08-00665-s001.pdf]

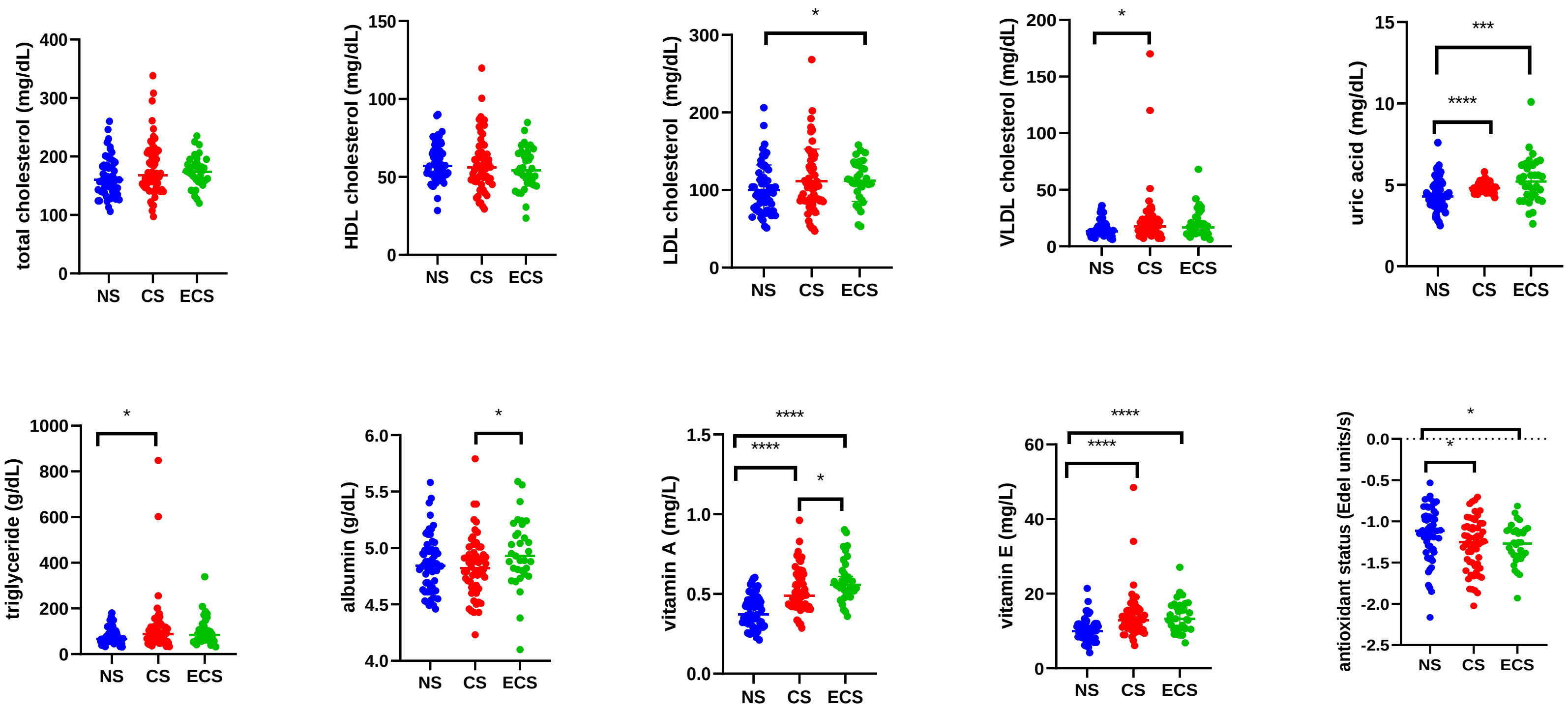

Figure 1. Biochemical results for tested samples. *P* values were calculated using Kruskal–Wallis test; where NS—Non-smokers; CS—Cigarette smokers; ECS—Electronic cigarette users; * $p < 0.05$; *** $p < 0.001$; **** $p < 0.0001$.

Table S1. Quantitative levels of hydrophobic components (total cholesterol, lipoprotein fractions, triglycerides, fat-soluble vitamins), albumins, and uric acid in serum of male non-smokers, cigarette smokers and e-cigarette users**.**

Parameter	Non-Smokers (n = 10)		Cigarette Smokers (n = 17)		E-Cigarette Users (n = 8)	
	**Mean ± STDEV**	**Median (p25th–p75th)**	**Mean ± STDEV**	**Median (p25th–p75th)**	**Mean ± STDEV**	**Median (p25th–p75th)**
Total cholesterol (mg/dL)	176.10 ± 38.76	175.50 (147.75–193.50)	191.24 ± 56.86	189.00 (147.00–222.50)	189.88 ± 21.45	183.00 (178.50–201.50)
HDL cholesterol (mg/dL)	51.18 ± 10.83	50.15 (43.60–56.13)	46.68 ± 13.66	45.00 (34.90–56.55)	44.23 ± 12.55	45.45 (32.98–55.10)
VLDL cholesterol (mg/dL)	16.30 ± 5.33	15.00 (11.75–22.25)	38.53 ± 42.46	24.00 (16.50–37.00)	27.38 ± 19.73	23.00 (11.25–39.00)
LDL cholesterol (mg/dL)	116.60 ± 40.28	115.00 (82.75–134.25)	118.82 ± 42.62	119.00 (87.00–151.00)	133.63 ± 12.95	136.00 (122.00–145.25)
Triglycerides (mg/dL)	83.60 ± 26.43	83.50 (57.75–113.25)	192.29 ± 212.33	121.00 (84.00–185.50)	136.13 ± 98.39	115.00 (56.75–193.75)
Vitamin A (mg/L)	0.46 ± 0.13	0.49 (0.32–0.57)	0.62 ± 0.15	0.65 (0.48–0.72)	0.65 ± 0.15	0.58 (0.52–0.79)
Vitamin E (mg/L)	10.05 ± 3.15	9.93 (7.30–11.78)	16.83 ± 10.20	15.08 (11.67–16.37)	15.44 ± 3.80	16.56 (11.07–18.64)
Albumin (g/dL)	5.12 ± 0.22	5.12 (4.93–5.32)	5.00 ± 0.34	4.93 (4.79–5.24)	5.10 ± 0.28	4.99 (4.88–5.36)
Uric acid (mg/dL)	5.44 ± 1.06	5.45 (4.60–6.03)	5.51 ± 1.34	5.40 (4.60–6.70)	6.80 ± 1.47	6.50 (5.70–7.20)

NS—Non-smokers (n = 10); CS—Cigarette smokers (n = 17); ECS—Electronic cigarette users (n = 8).

Table S2. Quantitative levels of hydrophobic components (total cholesterol, lipoprotein fractions, triglycerides, fat-soluble vitamins), albumins, and uric acid in serum of female non-smokers, cigarette smokers and e-cigarette users.

Parameter	Non-Smokers (*n* = 48)		Cigarette Smokers (*n* = 41)		E-Cigarette Users (*n* = 26)	
	**Mean ± STDEV**	**Median (p25th–p75th)**	**Mean ± STDEV**	**Median (p25th–p75th)**	**Mean ± STDEV**	**Median (p25th–p75th)**
Total cholesterol (mg/dL)	32.39 ± 47.82	11.85 (4.39–43.18)	39.35 ± 56.24	13.78 (5.14–53.65)	36.51 ± 48.00	12.44 (4.60–60.25)
HDL cholesterol (mg/dL)	34.07 ± 51.49	11.72 (2.63–45.80)	35.88 ± 47.74	15.25 (4.64–52.65)	45.30 ± 55.48	18.55 (4.63–86.07)
VLDL cholesterol (mg/dL)	32.00 ± 52.71	11.00 (2.92–45.75)	42.04 ± 60.62	14.37 (5.53–62.58)	38.79 ± 47.06	15.54 (4.56–80.50)
LDL cholesterol (mg/dL)	38.93 ± 67.75	10.80 (2.80–43.55)	39.39 ± 56.79	13.90 (4.67–56.00)	34.25 ± 42.93	12.66 (4.40–54.00) [(a)]
Triglycerides (mg/dL)	40.12 ± 80.82	11.19 (4.14–50.78)	39.03 ± 53.14	13.50 (4.44–54.00)	48.76 ± 60.16	17.35 (4.01–101.36) [(a)]
Vitamin A (mg/L)	36.51 ± 58.53	12.30 (2.42–47.75)	45.47 ± 61.15	14.51 (5.49–82.20)	43.82 ± 60.92	12.19 (4.74–69.50) [(a)]
Vitamin E (mg/L)	30.59 ± 48.37	10.20 (2.50–34.10)	42.64 ± 61.69	13.90 (5.20–62.00)	44.84 ± 56.05	16.42 (4.66–74.75) [(a)]
Albumin (g/dL)	31.48 ± 45.46	10.60 (3.37–45.25)	36.83 ± 49.33	13.10 (4.74–53.25)	49.19 ± 60.50	27.05 (3.94–84.25) [(a)]
Uric acid (mg/dL)	33.98 ± 50.53	13.00 (2.32–42.35)	49.91 ± 74.13	16.81 (4.80–51.23)	47.67 ± 57.74	18.68 (4.68–89.43) [(a)]

NS—Non-smokers (*n* = 48); CS—Cigarette smokers (*n* = 41); ECS—Electronic cigarette users (*n* = 26). [(a)]—one missing value (number of female ECS = 25)

Table S3. Quantitative levels of hydrophobic components (total cholesterol, lipoprotein fractions, triglycerides, fat-soluble vitamins), albumins, and uric acid in serum of cigarette smokers (CS) subgroups.

Parameters	Subgroup A (1–9 Cigarettes/Day) (*N* = 22)		Subgroup B (10–14 Cigarettes/Day) (*N* = 14)		Subgroup C (>15 Cigarettes/Day) (*N* = 18)	
	**Mean ± STDEV**	**Median (p25th–p75th)**	**Mean ± STDEV**	**Median (p25th–p75th)**	**Mean ± STDEV**	**Median (p25th–p75th)**
Age (years)	23.27 ± 7.38	21.00 (19.00–24.00)	27.64 ± 10.45	23.50 (20.50–37.75)	32.78 ± 10.89	30.00 (22.75–41.00)
Cigarettes/day	5.00 ± 2.14	5.00 (3.00–6.25)	10.21 ± 0.80	10.00 (10.00–10.00)	17.94 ± 2.46	20.00 (15.00–20.00)
Total cholesterol (mg/dL)	159.68 ± 35.31	152.50 (140.00–187.00)	193.93 ± 58.71	184.00 (150.75–227.25)	195.11 ± 50.00	183.50 (154.50–217.00)
HDL cholesterol (mg/dL)	55.55 ± 16.50	50.35 (46.70–65.88)	56.50 ± 16.73	56.20 (41.38–64.50)	62.07 ± 21.49	59.00 (48.75–70.60)
VLDL cholesterol (mg/dL)	17.09 ± 6.42	15.50 (12.00–20.75)	20.21 ± 10.26	19.00 (9.75–31.00)	25.00 ± 26.16	16.50 (11.00–30.25)
LDL cholesterol (mg/dL)	92.27 ± 28.53	87.50 (72.75–109.75)	127.36 ± 56.21	112.00 (86.00–155.50)	122.78 ± 39.91	117.50 (87.50–153.25)
Triglycerides (mg/dL)	85.49 ± 32.30	78.50 (61.00–104.00)	100.57 ± 51.56	93.50 (48.50–154.00)	125.11 ± 131.24	83.00 (55.75–150.00)
Vitamin A (mg/L)	0.51 ± 0.15	0.47 (0.42–0.60)	0.52 ± 0.14	0.49 (0.42–0.65)	0.50 ± 0.11	0.49 (0.42–0.62)
Vitamin E (mg/L)	13.59 ± 8.26	11.66 (10.26–14.01)	14.33 ± 3.66	14.80 (11.25–17.14)	14.31 ± 5.96	13.04 (10.84–15.47)
Albumin (g/dL)	4.84 ± 0.22	4.89 (4.69–4.94)	4.91 ± 0.28	4.92 (4.73–5.13)	4.82 ± 0.34	4.81 (4.53–4.94)
Uric acid (mg/dL)	4.83 ± 1.16	4.50 (3.98–5.73)	5.48 ± 1.26	5.25 (4.38–6.73)	4.30 ± 1.26	3.90 (3.33–5.50)

Obs. 4 persons didn't declare the real number of cigarettes per day.

Table S4. Quantitative levels of hydrophobic components (total cholesterol, lipoprotein fractions, triglycerides, fat-soluble vitamins), albumins, and uric acid in serum of e-users (ECS) subgroups**.**

	**Subgroup eA (1–9 Heets/Day) (*N* = 6)**		**Subgroup eB (10–14 Heets/Day) (*N* = 13)**		**Subgroup eC (>15 Heets/Day) (*N* = 15)**	
	**Mean ± STDEV**	**Median (p25th–p75th)**	**Mean ± STDEV**	**Median (p25th–p75th)**	**Mean ± STDEV**	**Median (p25th–p75th)**
Age (years)	31.67 ± 8.31	32.00 (24.75–38.75)	38.46 ± 10.09	42.00 (32.50–47.00)	33.80 ± 8.97	36.00 (25.00–40.00)
Cigarettes/day	5.17 ± 2.32	5.50 (2.75–7.25)	10.00 ± 0.00	10.00 (10.00–10.00)	18.67 ± 2.29	20.00 (15.00–20.00)
Total cholesterol (mg/dL)	171.17 ± 28.76	164.50 (151.75–188.25)	177.38 ± 15.14	180.00 (162.50–190.50)	170.47 ± 34.99	170.00 (141.00–203.00)
HDL cholesterol (mg/dL)	63.22 ± 12.89	66.35 (49.43–72.68)	53.19 ± 12.92	51.70 (46.85–64.15)	53.95 ± 14.29	53.30 (41.90–65.90)
VLDL cholesterol (mg/dL)	15.00 ± 10.16	12.00 (9.75–19.25)	18.00 ± 8.32	16.00 (11.50–21.00)	24.07 ± 15.29	20.00 (15.00–30.00) [(a)]
LDL cholesterol (mg/dL)	109.50 ± 20.71	107.50 (94.50–122.00)	120.92 ± 19.69	122.00 (107.25–136.75)	105.93 ± 32.53	109.00 (77.00–132.00) [(a)]
Triglycerides (mg/dL)	76.33 ± 50.96	61.00 (50.75–96.75)	89.92 ± 42.26	78.00 (57.50–106.50)	120.27 ± 76.27	99.00 (74.00–148.00) [(a)]
Vitamin A (mg/L)	0.55 ± 0.13	0.49 (0.47–0.70)	0.59 ± 0.14	0.55 (0.52–0.68)	0.60 ± 0.14	0.58 (0.52–0.65) [(a)]
Vitamin E (mg/L)	12.64 ± 3.14	11.80 (10.27–15.55)	16.76 ± 4.31	16.56 (13.54–19.08)	12.49 ± 3.54	11.56 (9.15–15.65) [(a)]
Albumin (g/dL)	5.05 ± 0.32	5.03 (4.74–5.32)	4.97 ± 0.38	4.98 (4.81–5.21)	4.91 ± 0.23	4.88 (4.77–5.11) [(a)]
Uric acid (mg/dL)	4.40 ± 0.41	4.30 (4.00–4.90)	5.38 ± 0.88	5.55 (4.48–6.15)	5.47 ± 1.90	5.40 (3.90–6.50) [(a)]

[(a)] one missing value (number of female from eB subgroup = 12).
